# Genetic diversity, temporal dynamics, and host specificity in blood parasites of passerines in north China

**DOI:** 10.1007/s00436-015-4695-5

**Published:** 2015-09-18

**Authors:** Xi Huang, Lu Dong, Chenglin Zhang, Yanyun Zhang

**Affiliations:** State Key Laboratory of Earth Surface Processes and Resource Ecology, Beijing Normal University, 100875 Beijing, China; College of Life Sciences, Beijing Normal University, 100875 Beijing, China; Department of Biology, Lund University, 22362 Lund, Sweden; Beijing Key Laboratory of Captive Wildlife Technologies, Beijing Zoo, 100044 Beijing, China

**Keywords:** Avian blood parasites, Temporal dynamic, Prevalence, Phylogeny, Lineage diversity

## Abstract

Avian blood parasites have been preliminarily studied in East Asia, but no data are available from long-term monitoring. The aim of this study was to evaluate the prevalence, genetic diversity, and temporal dynamics of *Plasmodium*, *Haemoproteus*, and *Leucocytozoon* in two passerine communities (one forest and one urban) in north China from 2008 to 2013, as well as the association between infected lineages and host specificities. Out of 633 birds from 40 species, 157 individuals (24.8 %) were infected; overall prevalence was 26.7 % and 16.8 % in two sites, respectively. The dominant avian blood parasite genus in the forest park changed yearly between *Plasmodium* and *Haemoproteus*, while the *Leucocytozoon* maintained a low infection level. Forty-four haplotypes were identified by sequencing a 432-bp fragment of the cytochrome b (*cyt b*) gene; more than 70 % were novel (six *Plasmodium* lineages, 16 *Haemoproteus* lineages, and nine *Leucocytozoon* lineages). Based on our data gathered over consecutive years, we found that the highly observed lineages of *Haemoproteus* showed higher host diversities than those of *Plasmodium*, and the most infected lineage EMEL01 (100 % identity with SGS1) take on the highest host diversity but low temporal diversity of the two genera, implying that this lineage infected a great diversity of species in certain years, but maintained a lower infection level or even disappeared in other years. The results suggest that genetic diversity of avian blood parasites in East Asia is high and provides scope for further research. In addition, compared with overall analysis, yearly prevalence monitoring is important in uncovering the temporal dynamic and host specificity variations over time.

**Electronic supplementary material:**

The online version of this article (doi:10.1007/s00436-015-4695-5) contains supplementary material, which is available to authorized users.

## Introduction

Understanding the prevalence and evolutionary pattern of parasites is of fundamental importance in basic research on both wildlife epidemic diseases and host–parasite co-evolution. Avian blood parasites, which include *Plasmodium*, *Haemoproteus*, and *Leucocytozoon*, are widespread, abundant, and easily sampled, making them excellent models for exploring the ecological and evolutionary dynamics of host–parasite associations (Valkiūnas 2004). Research on genetic diversity and infection patterns of avian blood parasites is of great importance when predicting wildlife diseases; therefore, it has gained considerable attention in recent decades (Riper et al. 1986; Scheuerlein and Ricklefs 2004; Valkiūnas 2004; Woodworth et al. 2005).

Long-term research on blood parasites in wild birds, which was mainly conducted in Europe, America, and Oceania, was helpful in detecting the key traits and variations in pathogens (Imura et al. 2012). Research on the temporal dynamic of avian blood parasites in the wild bird community can help to understand local changes and how lineages affect each other in the natural environment (Synek et al. 2013).

However, most long-term studies have so far aimed at revealing genetic diversity and infection features of samples collected in different years of one or a few host species, and limited data are available on temporal dynamics or lineage-based phylogenies. Temporal dynamics of lineage diversity can reflect interspecific interactions between parasites (de Roode et al. 2005). In previous research, the prevalence of three lineages (two *Plasmodium* and one *Haemoproteus*) showed significant variations over years in great reed warblers (*Acrocephalus arundinaceus*) (Bensch et al. 2007). Furthermore, a non-parallel annual variation in prevalence of two *Plasmodium* parasites, *Plasmodium relictum* and *Plasmodium circumflexum*, was reported in a population of blue tits (*Cyanistes caeruleus*) (Knowles et al. 2011).

Compared with the traditional method of blood smear screening, molecular-based screening has become much more popular in recent studies due to its high efficacy, accuracy, and ease of use (Møller et al. 2010; Valkiūnas et al. 2006). The use of molecular-based screening methods, combining with the traditional morphological characters (Valkiunas et al. 2007b, 2008), has enabled greater insight into avian parasite species diversity, enabling identification of species number from less than 200 (Valkiūnas 2004) to several thousand species (Beadell et al. 2006; Bensch et al. 2004). Although there is a great amount of avian diversity in China, the background information on avian blood parasites is very limited (Zehtindjiev et al. 2013; Zhang et al. 2014).

In order to detect the prevalence and temporal dynamic patterns of blood parasites, using molecular genetic screening techniques, we (1) present the genetic diversity and (2) detect the inter-annual change of the parasite lineages in passerine birds collected during 2008–2013 in Beijing, north China.

## Materials and methods

### Sample collection

A total of 633 passerines were captured with mist nets between September and October, from 2009 to 2013 at Xiaolongmen Forest Park (39° 57′ 54″ N 115° 26′ 00″ E) and 2008, 2012, and 2013 in the campus of Beijing Normal University campus (BNU, 39° 57′ 46″ N 116° 21′ 28″ E), Beijing. Blood samples were collected from the brachial vein and preserved in ethanol for subsequent processing in the laboratory. DNA was extracted using a TIANamp DNA kit (Tiangen, Beijing) according to the manufacturer’s protocol.

### Blood parasite identification

Blood parasites were identified using a nested PCR protocol based on the amplification of a 479-bp fragment of the mitochondrial cytochrome b (*cyt b*) gene (Hellgren et al. 2004). The first reaction included use of the primers HaemNFI (5′-CATATATTAAGAGAAITATGGAG-3′) and HaemNR3 (5′-ATAGAAAGATAAGAAATACCATTC-3′), performed in a 25 μL reaction mixture containing 12.5 μL 2× ExTaq buffer (Takara, Japan), 0.5 μL of each primer, and 1 μL template DNA. The second PCR reaction was carried out using 1 μL of the products amplified during the first reaction as template and included the primers HaemNF (5′-ATGGTGCTTTCGATATATGCATG-3′) and HaemNR2 (5′-GCATTATCTGGATGTGATAATGGT-3′). Positive or negative samples were determined by 1.5 % agarose gels stained with SYBR Green I in an ultraviolet trans-illuminator (GDS-8000PC, GENE, USA).

Nested PCR was conducted at least twice on each sample in order to avoid false-negative samples. Negative control (ddH_2_O) was used in each reaction. The PCR products of positive samples were sequenced in both directions using a 3730XL automatic sequencer (ABI, USA), and sequences were assembled by CodonCode Aligner 5.1.5 (CodonCode Corporation, USA).

### Phylogenetic analysis

Haplotypes identified by DnaSP 5.10.01 (Librado and Rozas 2009) and their most similar morphospecies, identified by the BLAST module in the MalAvi database (Bensch et al. 2009) were aligned by MEGA 5.04 (Tamura et al. 2011) for phylogenetic analysis. In addition, a fragment of *cyt b* sequence of the human malaria parasite *Plasmodium falciparum* (GenBank No.: M76611.1) was used as an out-group due to its close genetic relationship with avian haemosporidia (Outlaw and Ricklefs 2011). In total of four closely related morphospecies lineages to our results, SW3 (*Haemoproteus belopolskyi*) (Valkiunas et al. 2007a), ROBIN1 (*Haemoproteus attenuatus*) (Valkiūnas et al. 2006), YWT3 (*Haemoproteus motacillae*) (Dimitrov et al. 2013), and GRW06 (*Plasmodium elongatum*) (Valkiunas et al. 2008), identified by BLAST module in MalAvi database (Table 1), were employed as inner-group of phylogenetic analysis. Haplotypes with even one base difference from lineages in the MalAvi database were defined as new lineages and all sequences were submitted to Genbank (No.KT757541-KT757584).

**Table 1:** Summary of blood parasite lineages identified in passerines in Beijing, frequency of their identification in each sites, and BLAST result were shown according to the MalAvi database

Genus	Haplotype	Frequency	Frequency B^a^	Frequency X^b^	BLAST lineage	Identity
*Plasmodium*	EMEL01	31	0	31	SGS1	479/479 (100 %)
	PAPA01	1	0	1	SGS1	458/459 (99 %)
	EMGO01	2	0	2	GRW06	478/479 (99 %)
	EMGO02	1	0	1	SGS1	477/479 (99 %)
	EMEL02	6	0	6	ALARV04	479/479 (100 %)
	PAMO01	1	0	1	ALARV04	478/479 (99 %)
	PAPA02	1	0	1	ALARV04	418/420 (99 %)
	PHPR01	5	0	5	SYBOR02	433/433 (100 %)
	FIPA01	5	2	3	BT7	479/479 (100 %)
	ANHO01	1	1	0	BT7	474/475 (99 %)
	ANHO02	7	2	5	TURDUS1	479/479 (100 %)
	SATO01	1	1	0	EMSPO06	479/479 (100 %)
*Haemoproteus*	ACAE01	21	3	18	RW2	474/476 (99 %)
	PHPR02	2	0	2	RW2	476/479 (99 %)
	AECA01	2	0	2	RW2	475/479 (99 %)
	AECA02	2	0	2	RW2	458/461 (99 %)
	AECA03	16	4	12	FIPAR02	446/447 (99 %)
	EMEL03	19	2	17	HLW1	478/478 (100 %)
	MUDA01	1	1	0	ROBIN1	472/476 (99 %)
	AECA04	1	0	1	FIPAR02	462/464 (99 %)
	ANHO03	4	4	0	YWT3	477/479 (99 %)
	EMGO03	1	0	1	EMSPO01	479/479 (100 %)
	EMTR01	1	0	1	RW2	476/479 (99 %
	FIPA02	1	1	0	PAGRI04	419/431 (97 %)
	FIPA03	1	1	0	FIPAR02	459/464 (99 %)
	PAMA01	1	0	1	RW2	476/479 (99 %)
	PAMA02	1	0	1	RW2	476/479 (99 %)
	PHPR03	1	0	1	RW2	475/479 (99 %)
*Leucocytozoon*	EMPU02	1	0	1	TROAED09	470/478 (98 %)
	PAMA03	1	0	1	PYJOC02	440/450 (98 %)
	PAMA04	2	0	2	PYJOC02	467/476 (98 %)
	PAVE01	1	0	1	PYJOC02	394/404 (98 %)
	PAMO02	1	0	1	PYJOC02	419/428 (98 %)
	PAVE02	1	0	1	TRPIP2	416/422 (99 %)
	PAVE03	2	0	2	PARUS8	444/446 (99 %)
	PHIN01	1	0	1	PARUS8	418/430 (97 %)
	PHPR04	1	0	1	RS2	430/430 (100 %)
	SIEU01	1	0	1	HYBOR02	446/446 (100 %)
	PHPR05	4	0	4	BT5	383/383 (100 %)
	EMTR02	1	0	1	SBBS1	426/430 (99 %)
	FIPA04	1	1	0	SFC8	401/401 (100 %)
	TACY01	1	0	1	BT1	428/428 (100 %)
Undefined^c^	CAPU01	1	1	0	FULEU01	298/316 (94 %)
	EMPU01	1	1	0	SW3	300/315 (95 %)

^a^Frequency in the BNU campus (B)

^b^Frequency in Xiaolongmen Forest Park (X)

^c^These two haplotypes both showed the closest relationship with lineages belonging to *Haemoproteus* according to BLAST result, but clustered together with *Leucocytozoon* lineages in phylogenetic tree with low support

Nucleotide substitution models were tested using jModelTest 2.1.4 (Darriba et al. 2012), and corrected Akaike Information Criterion (AICc) was adopted so as to determine the best-fit model. BEAST v1.8.0 (Drummond and Rambaut 2007) was used for Bayesian phylogenetic inference with default parameters except for strict molecular clock and Yule process for tree prior. Markov chain Monte Carlo (MCMC) was run for different steps, with the length of the chain set as 1 × 10^9^ and log parameters as every 1 × 10^5^ generation. The maximum credibility tree was searched by TreeAnnotator v1.8.0 after the first 1000 trees were discarded as burn-in. The selected tree was then adjusted in FigTree v1.3.1 (Andrew Rambaut, University of Edinburgh, UK; http://tree.bio.ed.ac.uk/software/figtree/).

### Statistical analysis

Both the host species diversity index (S_H_) and temporal diversity index (T_S_) were calculated using the Shannon–Wiener index algorithm for lineages that infected at least four individuals. Considering the discontinuous sample collection in the campus, only samples collected in Xiaolongmen were utilized for analysis in this part.

$$ {S}_H=-{\displaystyle {\sum}_i^H\left({H}_i\right)\left({ \log}_2{H}_i\right)} $$

where *H* is the number of infected host species and *H*_*i*_ the proportion of infected individuals of species *i* (0 < *i* < *H*); then, supposing the number of infected individuals of the certain lineages is *N*, and in bird *i* is *n*_*i*_, then, *H*_*i*_ = *n*_*i*_/*N*.

$$ {T}_S=-{\displaystyle {\sum}_i^S\left({P}_i\right)\left({ \log}_2{P}_i\right)} $$

*S* represents the sample year, while *P*_*i*_ is the proportion of infected individuals in year *i* (0 < *i* < *S*). Supposing the number of infected individuals of the certain lineage is *N*, and in year *i* is *n*_*i*_, then, *P*_*i*_ = *n*_*i*_/*N*.

## Results

### Prevalence of avian blood parasites

A total of 633 birds belonging to 40 passerine species were sampled (Table S1), of which 157 individuals (24.8 %) were infected: 62 birds by 12 lineages of *Plasmodium*, 75 birds by 16 lineages of *Haemoproteus*; 19 birds by 14 lineages of *Leucocytozoon*, and two birds by two undefined lineages with low similarity (<95 %) to the reported lineages in MalAvi database. Only one willow tit, *Parus montanus*, showed an evidence of dual infection (both *Plasmodium* and *Haemoproteus* were detected in this sample). In Xiaolongmen Forest Park, 26.7 % (132/494) of the sampled individuals were infected including one dual infection. Among them, 35 lineages (10 *Plasmodium*, 12 *Haemoproteus*, and 13 *Leucocytozoon*) were detected. While in the campus, the overall prevalence was 16.8 % (25/139) with 14 lineages (four *Plasmodium*, seven *Haemoproteus*, one *Leucocytozoon*, and two undefined lineages).

**Fig. 1:**
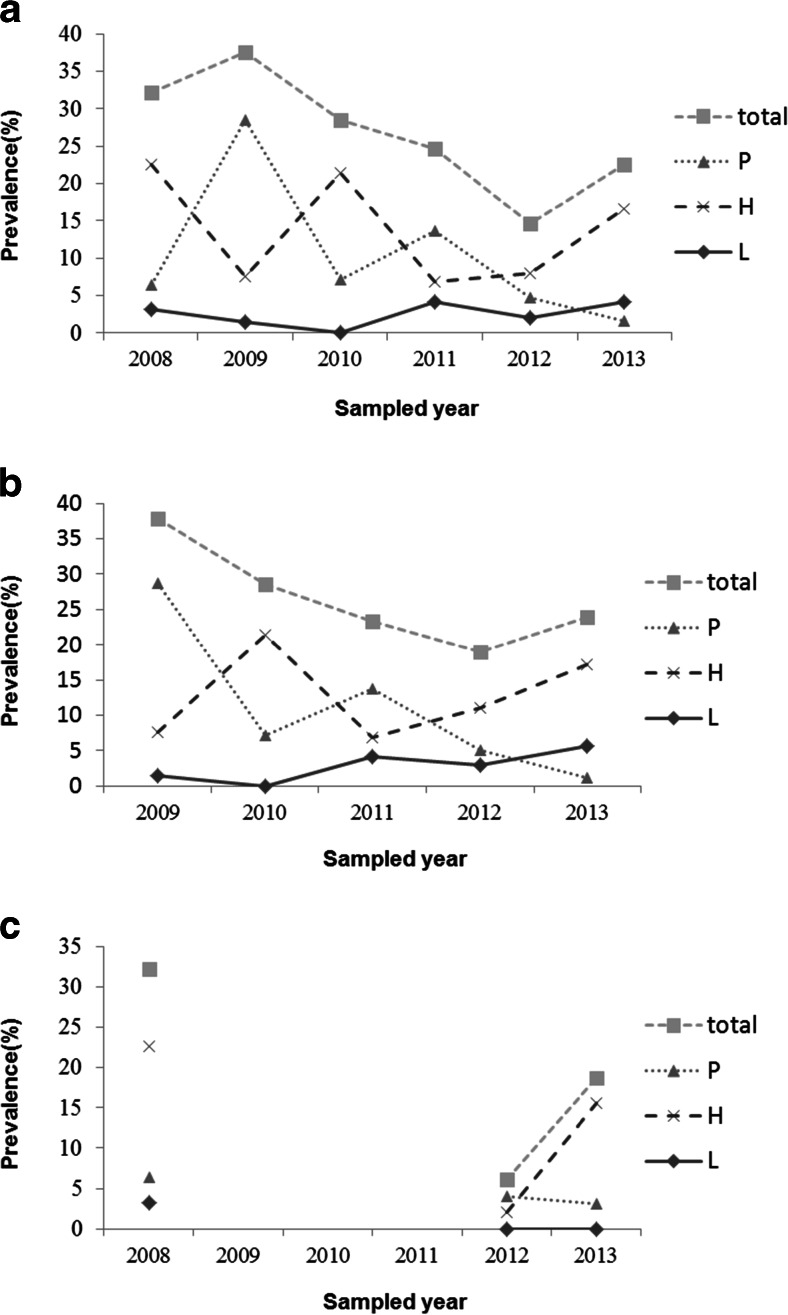
Annual variation (2008–2013) in prevalence of avian blood parasites in passerines in Beijing. **a** Total samples. **b** Xiaolongmen Forest Park. **c** BNU campus. *P Plasmodium*, *H Haemoproteus*, and *L Leucocytozoon*

The total prevalence was not considerably varied among years in Xiaolongmen Forest Park (*χ*^2^ = 8.01, *P* = 0.09), but the dominant parasite genus changed yearly between *Plasmodium* and *Haemoproteus* (Fig. 1) with significant variation among years (*Plasmodium χ*^2^ = 43.28, *P* < 0.01, *Haemoproteus χ*^2^ = 11.31, *P* < 0.05) and the prevalence of *Leucocytozoon* maintained a low level (0–4.17 %) with no significant differences over the years. In BNU campus, the significant yearly varied prevalence was observed in total samples (*χ*^2^ = 17.22, *P* < 0.01) and *Haemoproteus* (*χ*^2^ = 16.73, *P* < 0.01), but not significant in *Plasmodium* and *Leucocytozoon*.

### Phylogenetic tree

**Fig. 2:**
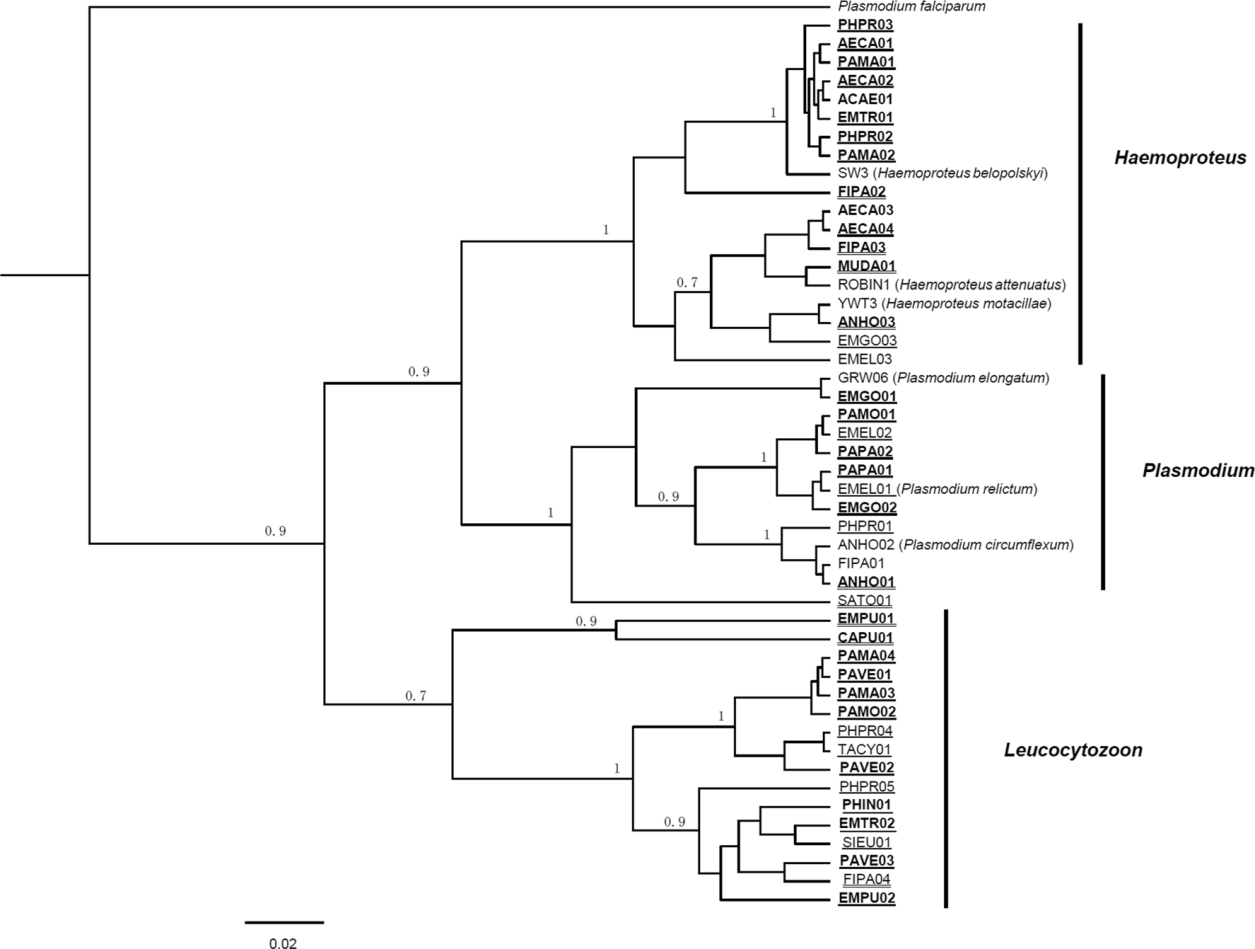
Bayesian *cyt b* phylogenetic tree of blood parasite haplotypes reconstructed under TN93 + I + G model. Posterior probabilities over 0.7 are listed above nodes. Novel lineages are indicated in *bold*. The lineages only detected in Xiaolongmen Forest Park were *underlined*, and only detected in BNU campus were* double* *underlined*. Morphospecies were labeled after the lineage in brackets

A total of 44 avian blood parasite lineages were detected in this study (Table 1), and 31 of them were novel. Two lineages could be designated as morphospecies according to BLAST in MalAvi database using cyt b sequences, EMEL01 (100 % with SGS1) belongs to *P. relictum* (Palinauskas et al. 2007) and ANHO02 (100 % with TURDUS1) belongs to *P. circumflexum* (Palinauskas et al. 2007). Three clades were formed in the Bayesian *cyt b* phylogenic tree (Fig. 2). All lineages of *Plasmodium* clustered together, forming a sister cluster to lineages of *Haemoproteus*. The lineages EMPU01 and CAPU01, which were most identified with *Haemoproteus* lineages according to the BLAST result, clustered together with *Leucocytozoon* lineages, although the node support was low (Fig. 2).

Most lineages that belong to *Plasmodium* are exactly the same as those reported ones. Morphospecies were combined with their most similar lineages respectively. The situation observed in *Haemoproteus* was much more complex with lineages differing from each other by only one or two bases and combined with the same morphospecies. *Leucocytozoon* lineages detected here are mostly novel.

### Lineage diversity and temporal dynamics

A total of eight lineages were identified as the most frequent in the wild passerine community in Xiaolongmen Forest Park (infecting at least four individuals), including four *Plasmodium* lineages, three *Haemoproteus* lineages, and one *Leucocytozoon* lineage (Table 2).

**Table 2:** Host diversity and temporal diversity of the eight most frequently detected lineages in Xiaolongmen Forest Park

Lineage	Genus	Host diversity		Temporal diversity	
		Host species^a^ (sample size)	*S*_*H*_	Sampled year (sample size)	*T*_*S*_
EMEL02	*Plasmodium*	Emel (1), Pamo (4), Pave (1)	1.25	2009 (1), 2010 (1), 2011 (1), 2012 (1), 2013 (2)	2.25
EMEL01	*Plasmodium*	Pama (3), Pamo (5), Pave (4), Emel (5), Emgo (3), Sieu (6), Papa (5)	2.77	2009 (25), 2011 (5), 2012 (1)	0.83
PHPR01	*Plasmodium*	Phpr (1)¸Tacy (4)	0.72	2009 (3), 2012 (2)	0.97
ANHO02	*Plasmodium*	Tacy (5)	0	2009 (4), 2011 (1)	0.72
AECA03	*Haemoproteus*	Pama (1), Tacy (9), Emel (1), Aeca (1)	1.21	2009 (5), 2012 (7)	0.98
EMEL03	*Haemoproteus*	Phin (2), Emel (7), Emgo (1), Fiel (1), Pama (2), Pamo (4)	2.51	2011 (2), 2013 (15)	0.59
ACAE01	*Haemoproteus*	Pama (5), Pamo (2), Tacy (1), Pave (2), Emel (1), Phpr (3), Sieu (3), Aeca (1)	2.77	2009 (3), 2010 (3), 2011 (3), 2012 (2), 2013 (7)	2.17
PHPR05	*Leucocytozoon*	Phpr (3), Tacy (1)	0.81	2011 (2), 2013 (2)	1.00

*S*_*H*_ host species diversity index, *T*_*S*_ temporal sampling diversity index

^a^Species codes correspond to species names in Table S1

Among the *Plasmodium* lineages, EMEL01 had the highest host species diversity index (*S*_*H*_ = 2.77) but the lowest temporal diversity index (*T*_*S*_ = 0.83), which suggested that it had an outbreak in a particular year (2009) and infected a diverse amount of bird species in the community, but then subsequently fell until disappearing in 2013. In contrast, EMEL02 had a higher temporal diversity index (*T*_*S*_ = 2.25) than others, implying that the prevalence of this lineage had shown little variation over the years. At the same time, ACAE01 had the highest value in both indexes (*S*_*H*_ = 2.77, *T*_*S*_ = 2.17) in *Haemoproteus*, showing a comparatively stable prevalence.

## Discussion

### Temporal prevalence dynamics of *Plasmodium* and *Haemoproteus*

We established the temporal dynamic of avian blood parasites in wild birds in north China across 6 years. Compared with similar fauna, the overall prevalence of avian blood parasites in northern China (26.7 % in forest and 16.8 % in urban) was comparable to that reported in western (Zehtindjiev et al. 2013) and southern China (Zhang et al. 2014), but was slightly higher than that reported in Japan (Murata 2002; Imura et al. 2012). The prevalence of blood parasites in passerine birds showed a significant variation over the years, as in other studies (Piersma and van der Velde 2012).

Ceratopogonid- and culicid-transmitted parasites, i.e., *Haemoproteus* and *Plasmodium*, showed a much higher prevalence than *Leucocytozoon*, which is transmitted by simuliid vectors. This is the opposite of the pattern in North America (Greiner et al. 1975) and Europe (Scheuerlein and Ricklefs 2004). As vector diversity and abundance are supposed to shape the diversity and the distribution of blood parasites (Santiago-Alarcon et al. 2012), further study of vector abundance in our study site would be helpful in pinpointing the cause behind this difference.

As reported previously, *Haemoproteus* seems to be more prevalent than *Plasmodium* (Silva-Iturriza et al. 2012). The generally accepted explanation is that of pathogenicity difference between the two genera (Atkinson et al. 2000). However, we discovered that *Haemoproteus* showed a higher prevalence in 4 of the 6 sampled years while the prevalence of *Plasmodium* was higher in the other 2 years. The different pattern uncovered here may due to the fact that the previous analysis of parasite prevalence and its contributing factors was based on pooled data from different years, while much less attention has been given to temporal dynamics (Ricklefs et al. 2005). Where both the biotic and abiotic factors and the correlation between parasites and their hosts may vary over different years (Baillie et al. 2012; Sol et al. 2003), this may induce temporal dynamics of annual prevalence of the two genera.

### Lineage diversity and temporal dynamics

Most of the lineages detected in this study merely infected one or two individuals (Table 1). Only six of them were found in both sites; the rest were mixed up in the phylogenetic tree, showing an unclear pattern between the forest and urban communities.

More than 70 % of the lineages were newly detected. Among them, CAPU01 and EMPU01 (both detected in Xiaolongmen) showed a large genetic divergence with lineages in the MalAvi database (only 94 and 95 % similar, respectively, with the closed reported lineages, Table 1) and held a unique position on the phylogenetic tree (Fig. 2), suggesting that they may represent a new species or new subgenus.

Within the 13 previously reported lineages, EMEL01 (SGS1), the most abundant one in *Plasmodium* as several previous studies in Europe, e.g., Ferrer et al. (2012), was detected almost all over the world (MalAvi database), while EMEL03 (HLW1), which was the most frequently detected *Haemoproteus* lineage in the present study, was only found in Asia (Bensch et al. 2000; Scordato and Kardish 2014).

The different patterns inside the main clade in the phylogenetic tree may be due to a different evolutionary history of the parasite genera. As postulated generalist parasites, *Plasmodium* spp. continuously infect novel hosts to form their evolutionarily stable associations (Beadell et al. 2004); therefore, lineages found in different host species may be exactly the same, and different species were clearly separated from each other. In contrast, *Haemoproteus* spp. have strong host–family specificities (Beadell et al. 2004). It is reported that *Haemoproteus* parasites are able to avoid hybridization within a host species although there are ample opportunities for mixing (Pérez-Tris et al. 2007), resulting in sympatric speciation, where new species were formed with slight differences.

The *Plasmodium* lineage EMEL01 had a high host diversity index, but showed a contemporarily low temporal diversity, implying that this lineage infected a great diversity of bird species in a certain year, but maintained a lower infection level and even disappeared in the following years. Meanwhile, the *Haemoproteus* lineages ACAE01 and EMEL03 break out suddenly or after several years’ stable prevalent respectively. Previous study has shown that there is a strong effect of competitive exclusion between *Haemoproteus* and *Plasmodium* (Szöllözi et al. 2011). Hence, EMEL01 may fade away with the eruption of ACAE01 and EMEL03.

Furthermore, it is reported that in great reed warbler populations, the chronic stages of infection was correlated with parasitemia during the acute phase (Asghar et al. 2012) and varied over the years (Asghar et al. 2011). If these findings are common in other species, they may also lead to a variation in prevalence.

It is generally accepted that *Plasmodium* lineages have a wider host range at the family level than other blood parasites. However, in our study, they did not show a higher host diversity compared with *Haemoproteus* lineages. This may be because our collection mainly focused on passerines, while *Haemoproteus* specificity is sometimes not obvious within an order, which was considered to be its maximum theoretical possible level (Valkiūnas 2004). Further study of more taxa from different orders is needed to address this question.

## Electronic supplementary material

Table S1 (DOCX 25 kb)
