# Supplementary material for: Genetic diversity, temporal dynamics, and host specificity in blood parasites of passerines in north China
Source: Parasitol Res. 2015 Sep 18;114(12):4513–20. doi: 10.1007/s00436-015-4695-5 (PMC4602065; doi:10.1007/s00436-015-4695-5)
Supplement: Supplementary file 1 — (DOCX 25 kb) [file 436_2015_4695_MOESM1_ESM.docx]

Supplementary Materials

Table S1. Information of sampled bird species and their infection lineages.

| **Species** | **Code** | **Sample size (X/B)**^*^ | **Numbers infected (X/B)** ^*^ | **Infection lineages** |
| --- | --- | --- | --- | --- |
| *Acrocephalus aedon* | Acae | 0/2 | 0/2 | ACAE01 |
| *Aegithalos caudatus* | Aeca | 37/0 | 5/0 | ACAE01, AECA01, AECA02, AECA03, AECA04 |
| *Anthus hodgsoni* | Anho | 3/21 | 0/5 | ANHO01, ANHO02, ANHO03 |
| *Carpodacus pulcherrimus* | Capu | 0/1 | 0/1 | CAPU01 |
| *Cyanopica cyana* | Cycy | 0/7 | 0/0 |  |
| *Emberiza cioides* | Emci | 2/0 | 0/0 |  |
| *Emberiza elegans* | Emel | 42/4 | 15/0 | ACAE01, AECA03, EMEL01, EMEL02, EMEL03 |
| *Emberiza godlewskii* | Emgo | 20/0 | 9/0 | AECA01, EMEL01, EMEL03, EMGO01, EMGO02, EMGO03 |
| *Emberiza pusilla* | Empu | 2/9 | 1/5 | ACAE01, AECA03, ANHO03, EMPU01, EMPU02 |
| *Emberiza rutila* | Emru | 1/0 | 0/0 |  |
| *Emberiza tristrami* | Emtr | 2/0 | 2/0 | EMTR01, EMTR02 |
| *Ficedula elisea* | Fiel | 1/0 | 1/0 | EMEL03 |
| *Ficedula parva* | Fipa | 0/15 | 0/8 | AECA03, FIPA01, FIPA02, FIPA03, FIPA04 |
| *Lanius cristatus* | Lacr | 0/1 | 0/0 |  |
| *Luscinia calliope* | Luca | 0/1 | 0/0 |  |
| *Luscinia svecicus* | Lusv | 0/1 | 0/0 |  |
| *Muscicapa dauurica* | Muda | 0/1 | 0/1 | MUDA01 |
| *Nucifraga caryocatactes* | Nuca | 1/0 | 0/0 |  |
| *Parus ater* | Paat | 1/0 | 0/0 |  |
| *Parus major* | Pama | 37/0 | 15/0 | ACAE01, AECA03, EMEL01, EMEL03, PAMA01, PAMA02, PAMA03, PAMA04 |
| *Parus montanus* | Pamo | 40/0 | 17/0 | ACAE01, EMEL01, EMEL02, EMEL03, PAMO01, PAMO02 |
| *Parus palustris* | Papa | 28/0 | 7/0 | EMEL01, PAPA01, PAPA02 |
| *Parus venustulus* | Pave | 29/0 | 12/0 | ACAE01, EMEL01, EMEL02, PAMA04, PAVE01, PAVE02, PAVE03 |
| *Passer montanus* | Pams | 0/15 | 0/0 |  |
| *Phoenicurus auroreus* | Phau | 3/0 | 0/0 |  |
| *Phylloscopus armandii* | Phar | 0/12 | 0/1 | EMEL03 |
| *Phylloscopus coronatus* | Phco | 3/0 | 0/0 |  |
| *Phylloscopus fuscatus* | Phfu | 9/1 | 0/0 |  |
| *Phylloscopus inornatus* | Phin | 23/11 | 3/1 | EMEL03, PHIN01 |
| *Phylloscopus plumbeitarsus* | Phpl | 0/1 | 0/0 |  |
| *Phylloscopus proregulus* | Phpr | 99/4 | 13/0 | ACAE01, AECA02, FIPA01, PHPR01, PHPR02, PHPR03, PHPR04, PHPR05 |
| *Phylloscopus reguloides* | Phre | 5/0 | 0/0 |  |
| *Phylloscopus schwarzi* | Phsc | 0/14 | 0/0 |  |
| *Phylloscopus yunnanensis* | Phyu | 0/1 | 0/0 |  |
| *Saxicola torquata* | Sato | 0/3 | 0/1 | SATO01 |
| *Sitta europaea* | Sieu | 19/0 | 10/0 | ACAE01, EMEL01, SIEU01 |
| *Tarsiger cyanurus* | Tacy | 90/4 | 23/0 | ACAE01, AECA03, ANHO02, FIPA01, PHPR01, PHPR05, TACY01 |
| *Urocissa erythrorhyncha* | Urer | 2/0 | 0/0 |  |
| *Urosphena squameiceps* | Ursq | 3/1 | 0/0 |  |
| *Zoothera dauma* | Zoda | 0/1 | 0/0 |  |

*X: Xiaolongmen Forest Park, B: Campus of Beijing Normal University
